# Involvement of Oxidative Stress and the Innate Immune System in SARS-CoV-2 Infection

**DOI:** 10.3390/diseases9010017

**Published:** 2021-02-24

**Authors:** Evgenii M. Kozlov, Ekaterina Ivanova, Andrey V. Grechko, Wei-Kai Wu, Antonina V. Starodubova, Alexander N. Orekhov

**Affiliations:** 1 Laboratory of Immunopathology, Department of Clinical Immunology and Allergy, Sechenov First Moscow State Medical University, 119991 Moscow, Russia; kozlov-evgeny@bk.ru; 2 Department of Basic Research, Institute of Atherosclerosis Research, 121609 Moscow, Russia; 3 Federal Research and Clinical Center of Intensive Care Medicine and Rehabilitology, 14-3 Solyanka Street, 109240 Moscow, Russia; noo@fnkcrr.ru; 4 Department of Medical Research, National Taiwan University Hospital, Taipei 10617, Taiwan; weikaiwu0115@gmail.com; 5 Federal Research Centre for Nutrition, Biotechnology and Food Safety, 2/14 Ustinsky Passage, 109240 Moscow, Russia; avs.ion@yandex.ru; 6 Pirogov Russian National Research Medical University, 1 Ostrovitianov Street, 117997 Moscow, Russia; 7 Laboratory of Angiopathology, Institute of General Pathology and Pathophysiology, 125315 Moscow, Russia; a.h.opexob@gmail.com; 8 Laboratory of Infectious Pathology and Molecular Microecology, Institute of Human Morphology, 117418 Moscow, Russia

**Keywords:** oxidative stress, SARS-CoV-2, innate immunity, macrophage

## Abstract

The emergence of the novel coronavirus in December 2019 in China marked the beginning of a pandemic that impacted healthcare systems and economic life all over the world. The virus primarily targets the respiratory system causing severe acute respiratory syndrome (SARS) in some patients, and therefore received the name of SARS-CoV-2. The pathogen stands out among other coronaviruses by its rapid transmission from human to human, with the majority of infected individuals being asymptomatic or presenting with only minor illness, therefore facilitating the pathogen spread. At the same time, people from the risk groups, such as the elderly, patients suffering from chronic diseases, or obese individuals, have increased chances of developing a severe or even fatal disease. The search for risk factors explaining this phenomenon continues. In this review, we focus on the known mechanisms of SARS-CoV-2 infection affecting the functioning of the immune system and discuss potential risk factors responsible for the severe disease course. Oxidative stress is one of such factors, which plays a prominent role in innate immunity activity, and recent research has revealed its tight involvement in SARS-CoV-2 infection. We discuss these recent findings and the development of excessive inflammation and cytokine storm observed during SARS-CoV-2 infection. Finally, we consider potential use of antioxidant drugs for alleviating the severe symptoms in affected patients.

## 1. Introduction

In December 2019, patients presenting with a novel respiratory disease caused by coronavirus, SARS-CoV-2, were reported in Wuhan, Hubei Province, China. The newly discovered virus was able to spread from human to human, and quickly led to a pandemic covering much of the world. By spring 2020, it became clear that the world healthcare and economic systems are facing a crisis never seen before. However, the coronavirus infection as such is not a novelty, and other coronaviruses are among known causes of human diseases.

The name “coronavirus” comes from the typical microscopic appearance of the viral particle, which has protruding spike proteins that resemble a crown. A member of coronavirus family HCoV-229E that affects bats and humans was first identified back in the 1960s. The pathogen is a single-chain RNA virus that interacts with aminopeptidase N, which serves as a virus receptor [1]. The HCoV-229E can infect humans causing upper respiratory tract disease, and is considered as one of the widespread causes of the human common cold. Since then, several other human coronaviruses have been detected and characterized. In 2003, a novel type of coronavirus, HCoV-NL63, was detected in the Netherlands in a 7-month-old child presenting with lower respiratory tract illness. The virus uses angiotensin-converting enzyme 2 (ACE2) as a receptor to enter the host cells. HCoV-NL63 was shown to be associated with moderate respiratory infection [2]. A powerful coronavirus outbreak that touched on more than 20 countries around the world occurred in 2002. The virus that caused it was called SARS-CoV because it caused severe acute respiratory syndrome (SARS). More than 8000 people were exposed to the virus during the outbreak [3]. Like HCoV-NL63, SARS-CoV uses ACE2 as a functional receptor [4]. The search for natural sources of the virus demonstrated that Egyptian bats can act as a reservoir for SARS-CoV, while intermediate hosts are civets, badgers, raccoon dogs, and other small mammals. Transmission from animals to humans has apparently occurred through consumption of badly cooked meat and inhalation of the waste products of bats. Because of the spread of SARS-CoV in numerous countries, the infection has acquired a mini-pandemic status [5]. In 2012, another type of coronavirus was isolated from a 60-year-old patient who died in a hospital in Saudi Arabia. The coronavirus caused the illness known as Middle East respiratory syndrome (MERS) and received the name of MERS-CoV [6]. The emergence of MERS-CoV caused much anxiety because it was associated with fatal lung failure reminiscent of SARS induced by SARS-CoV. The mortality rate in patients with MERS-CoV was estimated at 43%, which is a high rate, although the total number of MERS-CoV cases was only about 2500. MERS-CoV uses dipeptidyl peptidase 4 (DPP4) as its receptor [7].

A new dangerous coronavirus named SARS-CoV-2 was first described in the Chinese city of Wuhan in December 2019. The disease caused by the virus, COVID-19, is characterized by significant mortality and severity of symptoms, especially in at-risk populations, such as the elderly and individuals with comorbidities. Moreover, the virus can be easily transmitted between humans, including asymptomatic carriers. These factors helped COVID-19 become the most prominent of all coronavirus outbreaks detected so far, with over 10 million people worldwide already infected by the time this article was written, and this number continues to grow.

## 2. Structural Characteristics of SARS-CoV-2

SARS-CoV-2 is a β-coronavirus potentially mediating the development of SARS [8]. SARS-CoV-2 belongs to the order Nidovirales, family Coronaviridae, subfamily Orthocoronavirinae, genus Betacoronavirus and a species of coronaviruses provoking SARS [9]. It is an enveloped virus possessing continuous positive-stranded RNA varying from 26 kb to 32 kb in length. The diameter of SARS-CoV-2 viral particle can range from 70 to 90 nm [10].

Along with other members of coronaviruses, SARS-CoV-2 has four structural proteins, including Spike protein (S), Envelope protein (E), Membrane protein (M), nucleocapsid protein (N), together with 16 non-structural proteins (NSP) that are necessary for proper viral replication [11]. Given that SARS-CoV-2 is genetically similar to SARS-CoV, the virus structure data mapping was already largely available, allowing for relatively rapid characterization of the new pathogen [12].

Spike glycoprotein is a membrane-spanning protein that consists of two subunits and has the molecular mass of about 150 kDa. It forms homotrimers protruding outside of the viral particle and facilitates binding of the virus envelope with the host cells [13]. Once in the host cells, the S-protein is cleaved by furin-like protease (TMPRSS2) into two subunits: S1 and S2. The S1 subunit is responsible for binding with the host cellular receptor, and S2 subunit ensures the fusion of viral and cellular membranes [14].

The ACE2 protein, which is a functional receptor for SARS-CoV-2, is highly expressed in pneumocytes (type 2) of the respiratory epithelium [15]. The S1 subunit contains the N-terminal domain and receptor-binding domain (RBD), while S2 subunit is composed of the fusion peptide (FP), heptad repeats (HR), transmembrane domain (TM), and the cytoplasmic domain (CM) [16]. Despite the similarity of S-proteins between SARS-CoV and SARS-CoV-2, the affinity of SARS-CoV-2 S-protein to ACE2 is 10 to 20 times higher. That can be explained by the additional insert at the interface between S1/S2 sites, which can be cleaved by furin, whereby ligand-receptor interaction is increased, providing for a bigger opportunity for SARS-CoV-2 spreading [16,17,18].

The coronavirus N-protein is a highly conserved protein with a molecular weight of about 50 kDa. This structural component of SARS-CoV-2 participates in the processes of replication, transcription, and packaging of the viral genome. Moreover, it is involved in the cellular response of host cells to viral infection [19]. It was found that N-protein shares an amino acid homology of approximately 90% with SARS-CoV [20]. The interaction between RNA, N-protein, M-protein, and NSP3 leads to the formation of the ribonucleoprotein complex that is needed for virion assembly [21,22]. Structurally, N-protein consists of the N-terminal domain required for RNA binding, C-terminal domain necessary for oligomerization processes and a central Ser/Arg (SR)-rich linker, which has phosphorylation site and probably increases the affinity of N-protein to the viral RNA [23]. Given that N-protein is the most abundant coronavirus protein, and possesses a high immunogenic activity inducing the massive production of IgG and IgM, it can be potentially used for diagnostic purposes [24,25,26].

M-protein is a quite conserved integral protein among β-coronaviruses, which has a molecular weight of about 25–30 kDa. It is composed of three transmembrane-spanning domains connecting with a short N-terminal ectodomain and a long C-terminal endodomain [27,28]. M-protein was found to participate in the budding process, interaction with N-protein for stabilization of the nucleocapsid and the virion assembly facilitation [29,30].

One of the smallest coronaviral proteins is the E-protein, with a molecular weight of about 8–12 kDa [31]. This transmembrane protein has an N-terminal ectodomain, hydrophobic α-helical domain, and C-terminal hydrophilic endodomain. As has been seen, this protein takes part in the viral assembly, virions releasing, and it is implicated in the viral pathogenesis [32]. An important role of E-protein as viroporin has been demonstrated. Viroporins are viral proteins that have an impact on membrane permeability, ion flows, membrane remodeling, and glycoprotein traffic through forming a hydrophilic pore [33,34]. Consequently, E-protein is included in the pathogenesis of coronavirus infection through acting on the inflammasome formation and the release of Ca2+ from the Endoplasmic reticulum-Golgi intermediate compartment (ERGIC) [35]. In addition, viroporins are engaged in the assembly and release of virions from the infected cells, potentially through the disturbance of the chemoelectrical barrier and thus the membrane potential of the plasma membrane, which promotes the viral budding [34]. Moreover, a perturbation of ion homeostasis induced by viroporins may lead to apoptotic cell death [36].

## 3. SARS-CoV-2 Infection Is Associated with Oxidative Stress

Viral infection can affect cellular homeostasis, including the redox balance. Common viruses causing respiratory infections, such as human respiratory syncytial virus (RSV), influenza (IV), human rhinovirus (HRV), human metapneumovirus (HMPV), parainfluenza, adenoviruses and coronaviruses, usually cause only mild symptoms [37]. Presence of chronic respiratory diseases, such as asthma, chronic obstructive pulmonary disease, or mucoviscidosis, can influence the disease course by shifting the balance towards increased oxidative stress and reactive oxygen species (ROS) formation [38].

Oxidative stress is an imbalance between ROS production and the ability of the body to eliminate them by protective (antioxidant) mechanisms [39]. Under physiological conditions, ROS play a role as important signaling molecules which are capable of regulating the activity of enzymatic or transcription factors, therefore controlling metabolic processes [40]. It was shown that oxidative stress is associated with an increase of redox signaling, activation of transcription factors, stimulation activity of pro-inflammatory and pro-fibrotic cascades, DNA damage, and also stress-dependent kinases induction [41]. Furthermore, massive ROS generation (oxidative burst) is used by the immune cells to destroy the invading pathogens. Such excessive production of ROS can also have a direct destructive effect on the surrounding host cells, including the lung cells. That may be a possible mechanism inducing the severe lung pathology in SARS-CoV-2-infected patients [42,43]. Accordingly, the mechanisms underlying lung dysfunction may depend on the degree of oxidative stress manifestation being in close connection with the innate immune system activity.

The accumulating information on the clinical course of coronavirus disease shows that, so far, the majority of cases ascertained through testing appear to be asymptomatic or associated with only minor symptoms. At the same time, about 15% of the infected patients present with severe pneumonia, and 5% with multiple organ failure, toxic shock syndrome, and acute respiratory distress syndrome (ARDS) [44,45]. To date, the reason for such difference is not clear, but it is likely that the course of the individual’s immune response to the pathogen may play a role in it. The major cause of mortality associated with SARS-CoV-2 involves ARDS and associated so-called cytokine storm: an uncontrolled systemic inflammatory response provoking the expression of pro-inflammatory cytokines and chemokines by the effector cells [46]. The link between pro-inflammatory cytokine signaling and oxidative stress is being actively explored. For instance, it was shown that overexpression of pro-inflammatory cytokines can induce an increase in ROS generation [47].

Monocytes and macrophages are the key players of the innate immune response, which plays a prominent role in the inflammatory reactions associated with COVID-19 disease. These cells can release a number of pro-inflammatory cytokines, such as IL-1β, IL-6, IL-8, TNF, concentration of which may influence the severity of coronavirus infection. Therapeutic value of blocking the cytokine response is currently being studied [48]. It was found that patients with coronavirus infection presented with increased levels of circulating neutrophil extracellular traps (NET), indicative of neutrophil activation [49]. Moreover, high neutrophil proportion, which was observed in critically ill COVID-19 patients, was predictive of in-hospital mortality [50]. In their turn, neutrophils are among the main ROS producers. Antioxidant systems that evolved to alleviate ROS-associated damage in mammals are orchestrated by the expression of nuclear factor erythroid 2p45-related factor 2 (Nrf2) [51]. Under normal conditions, Nrf2 is retained in the cytoplasm by a protein cluster and rapidly degraded there. However, during oxidative stress, the factor is activated and stimulates a number of genes responsible for cytoprotection and detoxication. It was found that some viruses can suppress the Nrf2 pathway, therefore affecting the antioxidant response in the body. In particular, respiratory viral infections were shown to be associated with Nrf2 inhibition and activation of NF-kB pathway, leading to increased oxidative damage and promoting inflammation [37,50]. For instance, in RSV infection, which affects lower respiratory tract and is associated with respiratory insufficiency, ROS generation is induced in the epithelial cells of the respiratory system. This process is accompanied by transcription factor activation and cytokine and chemokine production. Moreover, RSV can induce lipid oxidation and decrease glutathione (GSH) concentration in alveolar type II-like epithelial cell line and inhibit the Nrf2 pathway, therefore reducing the expression of hemoxigenase-1 (HO-1), superoxide dismutase 1 (SOD1), superoxide dismutase 3 (SOD3), glutathione S-transferase (GST), catalase (CAT), and glutathione peroxidase (GPx) [52,53]. Moreover, a link was found between the decreased lung expression of SOD3 and COVID-19 severity in elderly people [54].

It is important to mention that the deteriorating effect of ROS is not limited to the respiratory epithelium, but affects other cell types, such as erythrocytes, which may contribute to the observed hypoxic respiratory failure in some patients with COVID-19 [55,56]. Hemolysis can increase the concentration of free heme and hemoglobin, which can further aggravate oxidative stress. Furthermore, elevated ROS generation affects the erythrocyte membrane, promoting phagocytosis in macrophages and neutrophils [52]. These processes are likely to contribute to the disease severity in COVID-19 patients and should be taken into account for future therapy development.

## 4. The Role of the Innate Immunity in Coronavirus Infection

Innate immune system is the first line of host defense that recognizes invading pathogens through sensing of pathogen-associated molecular patterns (PAMPs), such as foreign polysaccharides, glycoproteins, lipoproteins, and nucleic acids, as well as damage-associated molecular patterns (DAMPs) that include molecules generated as a result of damage of host cells and tissues. This task is performed by a variety of pattern recognition receptors (PRR) expressed by the innate immunity cells, such as monocytes/macrophages. Viral entry of the host cells induces the host immune response, which is initially mediated by antigen-presenting cells (APC); for example, dendritic cells (DCs) densely populating the respiratory tract [57]. Alveolar surface is a common site of encounter with bacterial and viral pathogens inhaled with air. Correspondingly, alveolar macrophages have a strong lytic potential, including the ability for oxidative burst [58]. Studies of coronavirus infection have shown so far that the initiation of the immune response to coronavirus typically begins in the respiratory epithelium. The APCs present in these sites express PRRs, such as toll-like receptor (TLR), nucleotide-binding oligomerization domain (NOD) like receptor (NLR) and retinoic acid-inducible gene (RIG) I-like receptors (RLR). Viral infection in the alveolae is therefore triggering PAMP recognition by the innate immune cells and the following initiation of the immune response [59].

TLRs are type I transmembrane glycoproteins that are expressed on both the immune cells (DCs, macrophages, B- and T-cells) and non-immune cells (fibroblasts, epithelial cells). Their extracellular part contains leucine-rich repeats (LRR) that mediate PAMP recognition, while the intracellular part contains a Toll/IL-1 receptor domain (TIR), which can interact with adapter molecules and trigger signaling pathways. In humans, 10 variations of TLRs are currently known. These receptors can be expressed both on the cell surface (TLR1, TLR2, TLR4, TLR5, TLR6, TLR10) and intracellularly (TLR3, TLR7, TLR8, TLR9) and are known to recognize different ligands. The cell surface TLRs bind to various components of the bacterial cell wall, such as lipids, bacterial lipopolysaccharide (LPS) and proteins, such as flagellin. The intracellular TLRs are able to recognize nucleic acids [59]. The recognition of SARS-CoV-2 by the innate immune system is being extensively studied. It was shown that the S-protein is recognized by TLR2 and TLR4, while viral genome fragments interact with TLR3 and TLR7/8 [60,61].

Two principal ways of TLR signaling transduction have been described: MyD88-dependent pathway (myeloid differentiation primary response protein 88) and TRIF-dependent pathway (TIR domain-containing adaptor-inducing IFNβ). Activation of MyD88-dependent pathway is associated with TIRAP binding (TIR domain-containing adaptor protein) followed by a myddosome complex formation that contains kinases of IRAK (IL-1R associated kinase) family [62]. This pathway activates nuclear factor-kappa B (NF-κB) and activator protein 1 (AP-1) transcription factors. Activation of the TRIF-dependent pathway occurs through recruitment of TRIF adaptor protein for the latter activation of TRAF (TNF receptor-associated factor) proteins, which in turn results in NF-κB and interferon regulatory factors (IRFs) activation.

NLRs are cytosolic receptors of innate immunity cells that play a key role in the regulation of the innate immune response. These receptors can induce the expression of pro-inflammatory cytokines in response to PAMP recognition, but also influence embryonic development, regulate apoptosis and participate in the reactions of the acquired immune system. NLRs are preferentially expressed in macrophages, DCs, and lymphocytes, but were also found in non-immune cells, such as epithelial cells. The spectrum of NLR ligands includes components of the bacterial cell wall, microbe toxins, DAMPs, and viral RNA. NLRs are large proteins containing 3 domains. In humans, as many as 22 members of the NLR family have been identified, differing by domain structure and functions. Based on a structural N-terminal domain organization, NLRs are divided into 4 subfamilies: NLRA, NLRB, NLRC, NLRP and also a single-member subfamily represented by NLRX1, a mitochondrial member of the NLR family [63]. The central domain of NLRs consists of 12 conserved motifs and is required for nucleotides and oligomerization (NOD/NACHT) binding. The N-terminal domain is variable, involved in protein–protein interactions, and has effector functions. This domain can contain caspase activation and recruitment domain (CARD), pyrin domain (PYD), acidic transactivation domain (AD) and baculovirus IAP repeat domain (BIR). The C-terminus contains LRRs that recognize PAMPs. Class II major histocompatibility complex transactivator (CIITA) and neuronal apoptosis inhibitory protein (NAIP) belong to the NLRA and NLRB families, respectively. CIITA of the NLRA family is a transcription regulator for the MHC II class, while NAIP, which belongs to the NLRB family, is involved in flagellin recognition, determination of type III secretion system components (T3SS), inflammasome formation and suppression of apoptosis [64,65,66]. The NLRC family is composed of 5 members: NOD1 (NLRC1), NOD2 (NLRC2), NLRC3 (NOD3), NLRC4 (IPAF), and NLRC5. Receptors of this family recognize many bacterial components mediating the host defense against bacterial infections. Moreover, the NLRC family is important for the tissue homeostasis and autophagy regulation [67,68]. The hallmark of this family of receptors is the presence of the CARD domain. Oligomerization of NOD1 and NOD2 leads to the recruitment of CARD-containing kinase RIP2 (receptor-interacting protein kinase) through the CARD–CARD interaction and further to nodosome formation and NF-κB activation [69]. Moreover, RIP2-kinase can induce mitogen-activated protein kinase (MAPK) pathway activation [70]. Activation of the abovementioned signaling pathways mediates synthesis of pro-inflammatory cytokines and the progression of the inflammatory response.

The NLPR family of receptors includes 14 members. The specific feature of this family is the presence of the N-terminal PYD domain that refers to the death effector domains (DED). It is known that DED takes part in apoptosis and the inflammatory processes. The members of the NLRP family, NLPR1, NLRP3, NLRP6, and NLRP7, were shown to form inflammasomes upon interaction with PAMP or DAMP. Inflammasomes are multimeric complexes that play a prominent role in the inflammatory response of the innate immune system [71]. Interaction of NLRPs with ligands induces receptor oligomerization and recruitment of ASC, apoptosis-associated speck-like protein, which contains PYD-CARD domains. Interaction between PYD domains of NLRP and ASC leads to the formation of polymeric filamentary structures and the recruitment of pro-caspase 1, which also contains a CARD [72]. Binding of CARD domains provoke the autocatalysis and activation of pro-caspase 1. In turn, active caspase 1 promotes generation of active forms of pro-inflammatory cytokines. The most studied inflammasome is NLRP3 (NOD-, LRR-, and pyrin domain-containing 3), also called cryopyrin (NALP3) [73]. Excessive activation of the NALP3 inflammasome is considered to be an essential factor during the initiation of cytokine storm and the following multiple organ failure in patients with COVID-19 [74]. Factors leading to NALP3 activation include nucleic acids, the cell wall components, DAMPs and several toxins which are capable of forming pores [75]. It was shown that viroporin E, ORF3a (open reading frame), and ORF8a are involved in the pathogenesis of the coronavirus infection and they are able to activate the NALP3 inflammasome via alteration of potassium efflux, cell volume, Ca^2+^ signaling, and lysosomes destruction [76,77,78].

RIG-I-like receptors are cytosolic proteins that participate in sensing the viral genome and the interferon synthesis for protecting from viral infection. Currently, 3 members of RLR have been defined: RIG-I, melanoma differentiation-associated protein 5 (MDA5), and laboratory of genetics and physiology 2 (LGP2), which belong to the DEAD-box protein family of RNA helicases. RLRs are abundantly expressed in myeloid, epithelial, and nerve cells. RLRs recognize viruses and execute antiviral activity through initiation of signaling cascades thanks to their specific structure. It was demonstrated that RLRs have 2 CARD domains on the N-terminus, the central RNA helicase core consisting of RecA-like Hel1 and Hel2 domains with the RNA-dependent ATPase activity and the C-terminus [79,80]. In the absence of viral components, RIG-I has a closed inactive conformation that is maintained by the C-terminus interacting with CARD. When PAMP is recognized by the helicase and the C-terminal domains, it leads to post-translational modifications performed by Riplet (RING-Type E3 Ubiquitin Transferase RNF135) and TRIM25 (tripartite motif-containing protein 25) proteins leading to RIG-I dimerization, conformational changes, and the release of the CARD domain. That allows for CARD–CARD interaction with the mitochondrial antiviral signaling protein (MAVS), which is located on the external mitochondrial membrane [79,81]. Binding of RIG-I/MDA5 with MAVS leads to the recruitment of the adaptor proteins mediating the activation of transcription factors. For example, recruitment of TRAF6 determines NF-κB activation while TRAF3 is responsible for IRF3/7 activation and follow-up induction of the interferon-stimulated genes, which help to restrict viral replication and promote antiviral response [82]. Therefore, RIG-I/MDA5 is considered to be a cytosolic sensor during coronavirus infection, which can recognize single-stranded RNA (ssRNA) and the intermediates of double-stranded RNA (dsRNA) in the course of SARS-CoV replication [83]. The role of these receptors can be demonstrated on the example of the immune evasion of SARS-CoV-2. It was noted that ORF9b interacts indirectly with Tom70 protein, causing suppression of MAVS signaling. That probably reflects the involvement of RLRs in the SARS-CoV-2 pathogenesis [84]. However, further studies are required for a detailed definition of PRRs contribution in the pathogenesis of COVID-19. Such investigations can potentially help finding some perspective targets for SARS-CoV-2 treatment.

Another group of proteins mediating antiviral response, which is especially important in the context of coronavirus infection, are interferons (IFNs), a group of signaling molecules. Two groups of IFNs can be distinguished: type I IFNs include IFN-α, IFN-β, and IFN-ω, and are also called viral IFNs, while type II IFN, IFN-γ, is also known as immune IFN. Type I IFNs are released in response to viral infection, and type II IFN—in response to mitogenic or antigenic stimuli [85]. Expression of different IFNs is triggered in response to PAMP-induced IRFs activation. Interaction of IFNs with their receptors leads to the activation of JAK-STAT (signal transducers and activators of transcription) pathway and the expression of IFN-stimulated genes that are involved in the antiviral response. However, coronaviral infection was shown to be associated with inhibition of the host’s antiviral response through certain mechanisms. It was found that SARS-CoV-2 can inhibit STAT phosphorylation, leading to reduced transcription of IFN-stimulated genes in monocyte-derived dendritic cells and macrophages [86]. Another study demonstrated that SARS-CoV-2 infection strongly inhibited type I and II IFN signaling through preventing nuclear translocation of STAT1 and 2, leading to reduced expression of IFN-stimulated genes. This anti-IFN activity was attributed to viral accessory protein Orf6, which is capable of interacting directly with Nup98-Rae1 factor, affecting the nuclear transport [87]. These mechanisms protect coronavirus against IFN-mediated host response, therefore increasing its pathogenicity.

## 5. Can Oxidative Stress Contribute to the Development of a More Severe SARS-Cov-2 Infection Course?

ROS are highly active agents that readily interact with and modify various molecules in cells and tissues, including nucleic acids, carbohydrates, and proteins [40]. Because of that, ROS are involved in both normal physiological and pathological reactions, and the increased levels of ROS are known to be associated with different human diseases. In human cells, ROS are produced mainly owing to the activity of diverse isoforms of NAPDH-oxidases through the electron leakage during the oxidative phosphorylation system (OXPHOS) by mitochondrial enzyme complexes which transfer electrons. In addition, the electron leakage is dependent on several enzymes such as xanthine oxidase, nitric oxide synthase (NOS), lipo- and cyclooxygenases, cytochrome P450, and activity of peroxisomes [88].

A prominent ROS-producing enzyme playing a key role in oxidative stress is nicotinamide adenine dinucleotide phosphate oxidase (NOX), which is a multi-subunit enzymatic complex. The family of NADPH-oxidases is represented by 7 members: NOX1, NOX2, NOX3, NOX4, NOX5, DUOX1, and DUOX2. All members of this family serve as electron carriers catalyzing the production of free radicals through reduction of molecular oxygen. Depending on the isoform, NOX proteins are expressed in different types of cells, such as epithelial, endothelial, smooth muscle, phagocytic cells, as well as in fibroblasts and osteoclasts [89]. NOX2 is the first described enzyme of this family which was discovered on the phagosomal membrane. This isoform is composed of integral gp91phox (β-subunit) and p22phox (α-subunit) proteins that together form a large heterodimeric Cyt b558 subunit. The p40phox, p47phox, p67phox subunits are cytosolic components and activation signals are required for their translocation toward the plasma membrane and further assembly of the complex [90]. Formation of active NADPH-oxidase requires phosphorylation of p47phox through PKC (protein kinase C), which leads to the assembly of the triple complex and its translocation toward the membrane [91]. Moreover, GTPase Rac1/2 is required for a proper NOX activity. Rac1/2 and p67phox are catalytic subunits that mediate the electron transfer from NADPH to the prosthetic group of gp91phox (FAD and heme), and eventually, electrons transmission to molecular oxygen and superoxide ion formation [92].

Cytokine storm is a life-threatening condition associated with uncontrolled cytokine expression, observed in some severe human disorders, including severe cases of COVID-19. The main representatives of the innate immune system in the airways are epithelial cells, macrophages, and DCs which detect the viral particles and trigger the inflammatory cascades [57]. Because SARS-CoV-2 has a tropism to the airway epithelium, the expression of cytokines, chemokines, and cell adhesion molecules is induced by the virus-infected cells. Generation of these molecules gives rise to the signals for attracting more immune cells which mediate tissue damages and further amplification of the inflammatory response [93]. Hence, cytokines appear to be among the most important molecules during SARS-CoV-2 infection. At the same time, activation of PRRs and production of the pro-inflammatory cytokines is a substantial factor for oxidative stress development. For instance, it was found that TLR signaling can be involved in NOX priming, activation and translocation towards the plasma membrane by NOX subunits phosphorylation [94]. Thus, TLR signaling is an influential mechanism promoting NADPH-oxidase assembly, and therefore accelerating ROS production. Apart from this, it was shown that activation of NF-κB signaling increases the expression of the gp91 subunit [95]. The increase of ROS concentration, as well as decrease of antioxidant defense leads to oxidative stress development [41]. It is worth mentioning that ROS not only mediate direct ROS-induced oxidative damage of biomolecules, but also participate in redox signaling, influencing the activity of transcription factors, kinases, caspases, receptors through post-translational modifications [96]. Moreover, ROS can perform a double role during oxidative stress. On one hand, they are able to activate NF-κB pathway that leads to the induction of the pro-inflammatory cytokines and cell adhesion molecules (ICAM-1, VCAM-1, P- and E-selectin) expression on the endothelial cells, where they facilitate interaction with lymphocytes. That is especially important during ARDS emergence, when polymorpho-nuclear neutrophils are one of the main ROS producers [39,43,97,98]. On the other hand, ROS have the ability to repress of NF-κB signaling through an adaptive mechanism for decreasing the severity of inflammation and oxidative stress [95]. Interestingly, NF-κB activation may induce synthesis of different antioxidant proteins that can reduce the concentration of free radicals as well [99].

There are several other factors that can amplify oxidative stress in the body during SARS-CoV-2 infection. For example, it is well known that electron leakage is observed during OXPHOS in I and III mitochondrial complexes that lead to the formation of mitochondrial ROS (mtROS) and stimulate gradual mitochondrial damage [100]. It is supposed that mtROS together with oxidized mitochondrial DNA induce NALP3 activation [101]. Mitophagy is a process for degradation of damaged or dysfunctional mitochondria that allows maintaining of functional population of these organelles in the cell. Certain nuclear and mitochondrial DNA mutations can suppress mitophagy, which was found to be associated with increased pro-inflammatory response and inflammation chronification [102]. Mitochondrial dysfunction and deficient mitophagy are present in various human chronic diseases. It is possible that presence of mitochondrial dysfunction coupled with oxidative stress contributes to the increased severity of SARS-CoV-2 infection. Several other conditions are known to be associated with increased oxidative stress as well. Obesity is known to be one of them. It was found that accumulated adipose tissue produces the pro-inflammatory cytokines, thereby provoking ROS generation [103,104].

Smoking is another factor that greatly contributes to oxidative stress. It was demonstrated that cigarette smoking impacts the endothelial functions, intensifies oxidative stress, and causes platelet activation [105]. Moreover, ACE2 expression is increased in response to lung injure and inflammation caused by smoking [106]. Together, these factors may explain the observed more severe course of SARS-CoV-2 infection in smokers. At the same time, several groups have reported the so-called “smoker’s paradox”: the fact that smokers appear to be underrepresented among COVID-19 patients in some populations. Epidemiological and case-control studies reported that the prevalence of active smokers among hospitalized COVID-19 patients was lower than expected, but the reasons for this phenomenon remained unclear [107]. The existing hypotheses include blunted immune response in active smokers, elevated nitric oxide in the smokers’ respiratory tract, and anti-inflammatory effects of nicotine. However, the data available so far are quite limited, and not free from biases. Moreover, the deteriorating effect of smoking on the respiratory tract is likely to aggravate the disease severity [108]. More systematic studies are needed to explore the “smoker’s paradox”, which should be treated with caution until solid data are obtained.

Older age is another factor associated with increased oxidative stress, since the protective capabilities of the antioxidant systems of the body are generally reduced in older adults [109]. That can be associated with aggravated pro-inflammatory response that leads to ARDS development [110]. Therefore, several factors that are known to be associated with increased oxidative stress, such as chronic diseases, including morbid obesity and diabetes mellitus, smoking, and older age, were also listed as risk factors for a more severe course of COVID-19 [111].

**Figure 1:**
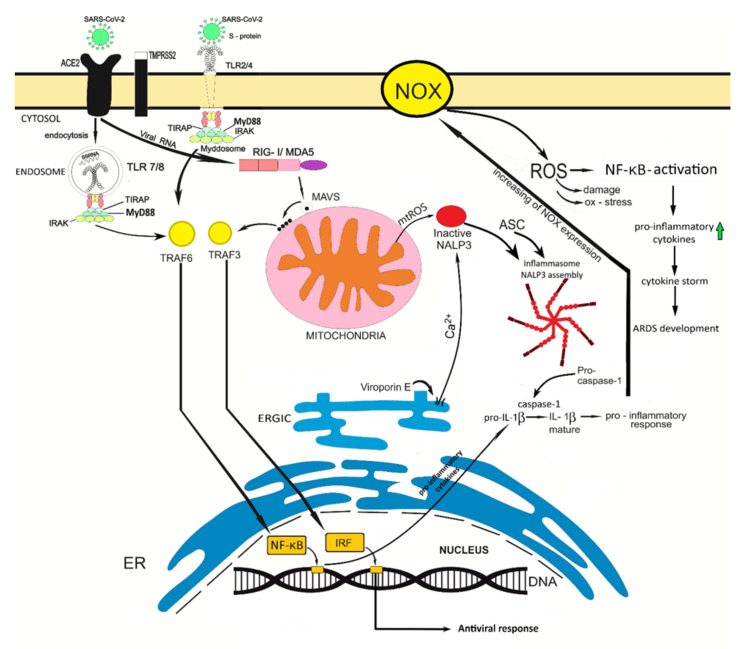
Possible ways of oxidative stress involvement in the COVID-19 disease. SARS-CoV-2 binds with ACE2 receptor; as a result, this virus enters into the host cells by endocytosis. The different virus components are recognized by PRR. For example, S-protein is distinguished by TLR2/4; ssRNA of coronavirus is distinguished by TLR7/8. Besides, RIG-I/MDA5 could identify dsRNA, a replication intermediate for RNA viruses. When sensing of dsRNA is performed, it leads to the cooperation with MAVS and further oligomerization process that attracts the adaptor proteins with subsequent activation of NF-κB and IRF factors. Also, viroporin E is capable of changing the ion flows of Ca^2+^ and K^+^ which drive to NALP3 activation. TLRs sensing provides the recruitment of adaptor proteins which can form the myddosome complex for NF-κB activation. NF-κB signaling is linked by expression of interleukin precursors. The activation effects on NALP3 inflammasome are necessary to realize the olizomerization process and ASC recruitment. Eventually, the formed inflammasome converts pro-caspase-1 to active caspase-1 in order to execute the processing of pro-interleukins. Stimulation of NF-κB signaling is associated with increasing of NOX expression, succeeded by ROS production. ROS can serve as signaling molecules that induce NF-κB signaling as well. Thus, the excessive ROS production mediates the hyper-inflammation and development of cytokine storm that impact both the possibility of ARDS development and severity of ARDS course.

The information on the possible involvement of oxidative stress in the pathogenic mechanisms of SARS-CoV-2 infection is constantly accumulating. The information available to date is presented in Figure 1. The search for old and new medications that can alleviate the symptoms of the disease and reduce mortality is currently a top priority, and antioxidant drugs should be considered as well. A potential agent which can decrease oxidative stress for SARS-CoV-2 patients is N-acetylcysteine (NAC). It is a precursor of glutathione (GSH), which in turn serves as the most important regulator of intracellular redox potential. Moreover, NAC is able to inhibit NALP3 inflammasome that lowers the inflammatory response and ROS-mediated synthesis [112]. Furthermore, clinical guidelines for COVID-19 treatment suggest using NAC, vitamins E/C and melatonin. Melatonin is considered to be a powerful antioxidant because it is capable of scavenging a wide pool of free radicals and has an anti-inflammatory effect which increases the expressions of antioxidant enzymes [113]. Finally, an animal model of ARDS was used to demonstrate alpha-lipoic acid and vitamins E/C using for increasing the GSH level and the decline of TNF-α (tumor necrosis factor) and IL-1β concentration. Thus, antioxidant therapy is necessary for reducing the severity of SARS-CoV-2 and its applying can prevent ARDS development [114]. Further studies will undoubtedly improve our understanding of the potential of antioxidant drugs for treatment of COVID-19 disease.

TLR, toll like receptors; ACE2, angiotensin-converting enzyme 2; ssRNA, single-stranded RNA; dsRNA, double-stranded RNA; MAVS, mitochondrial antiviral-signaling protein; IRF, interferon regulatory factor; ASC, apoptosis-associated speck-like protein containing a CARD; ROS, reactive oxygen species; NOX, NADPH-oxidase.

## 6. Conclusions

The SARS-CoV-2 pandemic has become a great challenge for the world community and has already led to more than 700 thousand deaths all over the world. However, the disease severity and mortality vary widely across populations, and it is now clear that certain risk factors should exist that explain such substantial differences. Some patients develop ARDS, which is preceded by a cytokine storm, which in turn is associated with virus detection by PRRs and subsequent hyperactivation of the NALP3 inflammasome. Future investigations should put special emphasis on PRRs that mainly participate in virus recognition. Currently available data on receptors for SARS-CoV-2 detection are limited. The known risk factors of severe disease course and mortality from SARS-CoV-2 infection include a number of chronic diseases, such as diabetes, obesity, and older age. Interestingly, the list of these factors overlaps with the known list of human conditions associated with increased oxidative stress. Certain signaling pathways of oxidative stress can have a direct influence on cytokine storm triggering during the infection. Therefore, the impact of ROS on the severity of coronavirus infection is an important question and should be investigated by future studies. Appropriate antioxidant therapy will probably make a significant contribution to decreasing the hyperinflammation rate as well as reducing the severity of ARDS.
